# Evaluation of the Antibacterial Properties of Polyvinyl Alcohol-Pullulan Scaffolds Loaded with *Nepeta racemosa* Lam. Essential Oil and Perspectives for Possible Applications

**DOI:** 10.3390/plants12040898

**Published:** 2023-02-16

**Authors:** Constantin Lungoci, Cristina Mihaela Rîmbu, Iuliana Motrescu, Diana Serbezeanu, Cristina Elena Horhogea, Tăchiță Vlad-Bubulac, Carmen Simona Ghițău, Ioan Puiu, Andra-Sabina Neculai-Văleanu, Teodor Robu

**Affiliations:** 1 Department of Plant Science, Iasi University of Life Sciences, 3 Sadoveanu Alley, 700490 Iasi, Romania; 2 Department of Public Health, Iasi University of Life Sciences, 8 Sadoveanu Alley, 707027 Iasi, Romania; 3 Department of Exact Sciences, Iasi University of Life Sciences, 3 Sadoveanu Alley, 700490 Iasi, Romania; 4 Research Institute for Agriculture and Environment, Iasi University of Life Sciences, 9 Sadoveanu Alley, 700490 Iasi, Romania; 5 Department of Polycondensation and Thermally Stable Polymers, “Petru Poni” Institute of Macromolecular Chemistry, 41A Grigore Ghica Voda Alley, 700487 Iasi, Romania; 6 Research and Development Station for Cattle Breading Dancu, 9 Iasi-Ungheni rd., 707252 Dancu, Romania

**Keywords:** *Nepeta*, essential oil, antimicrobial activity

## Abstract

Essential oil of *Nepeta racemosa* Lam. was extracted and characterized to determine its antimicrobial activity and potential use in applications. The essential oil was loaded on polyvinyl alcohol-pullulan films and gels and characterized by optical microscopy, scanning electron microscopy, and UV-Vis spectroscopy before having its antimicrobial capacities assessed. The essential oil extracted from *Nepeta racemosa* Lam. was characterized using gas chromatography coupled with mass spectroscopy, which indicated that the most abundant component was nepetalic acid (55.5%), followed by eucalyptol (10.7%) and other compounds with concentrations of about 5% or less. The essential oil, as well as the loaded films and gels, exhibited good antibacterial activity on both gram-positive and gram-negative strains, with growth inhibition zones larger in some cases than for gentamicin, indicating excellent premises for using these essential-oil-loaded materials for applications in the food industry or biomedicine.

## 1. Introduction

Antimicrobial resistance represents one of the top 10 global public health threats according to the World Health Organization [1]. The impact on humanity is immense from many points of view—medical, social, economic, environmental—with many drug-resistant bacteria, viruses, and fungi, and therefore an urgent need for overcoming some of these issues [2,3]. Good candidates are found among3 plant-based phytochemicals that have proved to have good antimicrobial properties used by themselves or when complementing antibiotics or other medicines [4,5,6,7,8,9,10,11,12,13,14,15]. 

The experiment is a part of a more complex project with the purpose of following the initial concept proposed by the European Commission: "From farm to fork” [16]. In particular, this concept is represented by the acclimation of new species such as *Nepeta racemosa* Lam. [17], testing them in different technological conditions [18], or under the influence of different abiotic factors [18,19,20], processing them to obtain products with antimicrobial activity. Plant extracts have generated substantial interest in the food industry, since there is a need to avoid the use of harmful artificial chemicals such as food preservatives. To this extent, essential oils and other secondary metabolites extracted from plants have been studied in the past couple of years for their antioxidant and antimicrobial properties, and as potential preservatives of flavors and fragrances to be used in foods [21,22,23,24,25]. These essential oils can be applied directly to the fresh produce as sanitizers or can be integrated in the containers in smart packaging materials [26,27,28,29,30,31,32]. The antimicrobial activity of such novel materials could also benefit other applications such as medical technologies; in this case, tissue-engineering-produced scaffolds as cell support need both bioactive and antibacterial characteristics [33]. 

Biologically active antibacterial scaffolds have been studied extensively as the basis for tissue engineering, or as matrixes with drug delivery capacities [34]. Polymeric materials with antibacterial properties have also been used in food packaging, textiles, and other applications where the proliferation of bacteria is unwanted [35,36,37,38,39,40,41,42]. The present investigation aims to incorporate the bioactive compounds present in *Nepeta racemosa*, an important ethnomedicinal plant, into a polymer matrix with the aim of using these new scaffolds in food packaging, biomedical fields, or in phytomedicine, based on their antimicrobial property. To achieve our goal, new scaffolds were fabricated based on polyvinyl alcohol (PVA), which is an FDA-approved synthetic polymer that can be used as a substitute for polar and water-soluble material [43], pullulan, which is a polysaccharide, and *Nepeta racemosa* Lam. essential oil (EO). Due to its intrinsic properties, such as advanced water solubility and facile biodegradability, PVA is also known as a “green polymer” [44], exhibiting excellent biocompatibility, non-toxicity, film and gel forming capacities [45,46]. In the pharmaceutical industry, PVA-based hydrogels are used as delivery systems for various bioactive compounds [47]. Pullulan has also biodegradable, biocompatible, and non-toxic properties [48], and was used in combination with PVA to modulate the tunable properties of the desired composites [49]. Composite hydrogels based on PVA and pullulan were used for tissue engineering applications [50], as well as for protective packaging for food and pharmaceutical products [51,52,53]. To obtain scaffolds with remarkable properties, two series of PVA–pullulan composites were proposed, one of a series containing PVA–pullulan-based films loaded with essential oil extract, and a second series of PVA–pullulan, chemically crosslinked with oxalic acid loaded with essential oils extract. The prepared scaffolds were characterized to confirm the presence of the plant extract on their surface, and their antimicrobial activity was assessed. 

## 2. Results

### 2.1. Characterization of Nepeta racemosa Lam. Essential Oil

The content of essential oil was determined in the inflorescence emergence phase, at the first harvest of *Nepeta racemosa* Lam., which takes place in the first week of May. From the extraction, 0.35 ± 0.08 % essential oil (EO) was obtained. The main component identified in the GC-MS analysis was nepetalic acid (55.5%), followed by eucalyptol (10.7%) and 3-Hydroxy-(4S,4aS,7S,7aS)-Dihydronepetalactone (5.4%). The other components found had a much smaller presence, between 0.1–5% (Table 1).

### 2.2. UV-VIS Spectroscopy Analysis 

**Figure 1:**
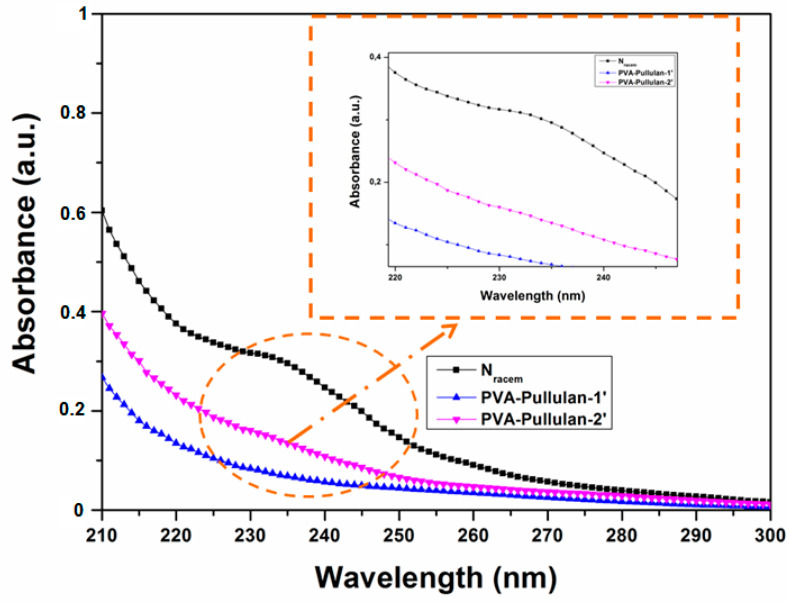
UV-VIS spectra of *N. racemosa* EO and *N. racemosa* EO-loaded PVA–pullulan gels.

In order to demonstrate the incorporation of *N. racemosa* EO into the PVA–pullulan polymeric matrices and also the release capacity of *N. racemosa* EO from the dry gels, PVA–pullulan-1 and PVA–pullulan-2 gel samples were extracted with ethanol and the resulted solutions were subjected to UV-Vis spectroscopy analysis. The results of the UV-Vis spectra analysis are presented in Figure 1. The investigation of the pure *N. racemosa* EO revealed a shoulder in the range of 220–240 nm. In the case of the extracted solutions from loaded PVA–pullulan, the *N. racemosa* EO shoulder appeared diminished in the case of PVA–pullulan-1 extract, yet, its presence became more evident in the case of PVA–pullulan-1 extract, as the inset from Figure 1 revealed, suggesting that a higher loading of EO into the PVA–pullulan crosslinked network will result in a higher degree of *N. racemosa* EO release from these types of polymeric matrices. 

### 2.3. Fourier Transform Infrared Spectroscopy (FTIR)

**Figure 2:**
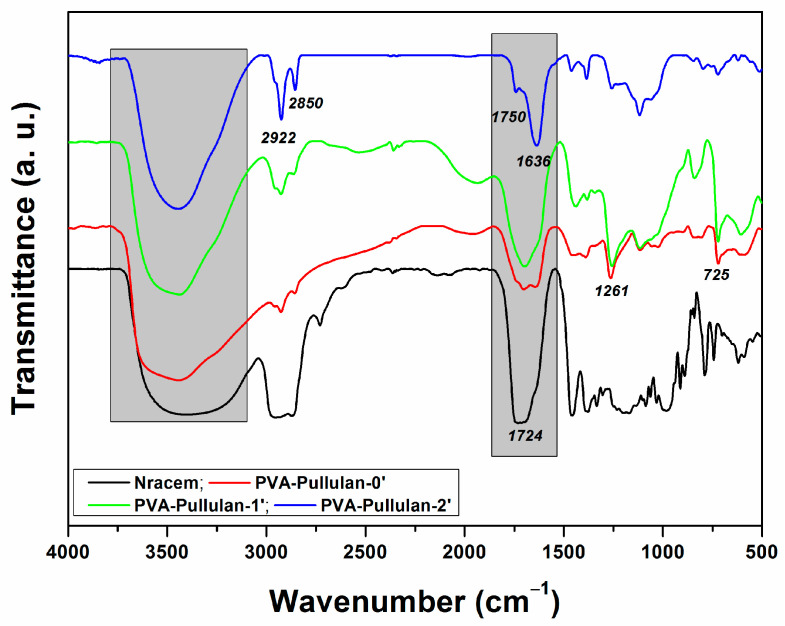
FTIR spectra of the PVA–pullulan loaded with *N. racemosa* EO.

FTIR spectra for *N. racemosa* EO and for the PVA–pullulan reinforced with oxalic acid are presented in Figure 2. The absorption band appearing at 850 cm^−1^ is characteristic of the a-glucopyranoside units, while the absorption band at 725 cm^−1^ indicates the presence of α-(1,4) glucoside bonds. Furthermore, the characteristic absorption tiny band located at 932 cm^−1^ proves the presence of α-(1,6) glucoside bonds existent in the pullulan molecule. Bands at 2850–3000 cm^−1^ are caused by stretching vibrations of the CH and CH_2_ groups, while bands at 1300–1500 cm^−1^ are caused by deformation vibrations of the CH and CH_2_ groups. A very intensive, broad hydroxyl band occurs between 3000–3600 cm^−1^, and accompanying C–O stretching vibrations were revealed at 1000–1260 cm^−1^. Alternatively, in the FTIR spectra of *N. racemosa* EO-loaded samples, peaks at 1743 cm^−1^, 1372 cm^−1^, 1240 cm^−1^, 1157 cm^−1^, and 1101 cm^−1^ were observed. These peaks corresponded to flavanols, flavanone, chloro-flavones, ursolic acid, tannins, and flavones, respectively, and were attributed to the characteristic vibrations of active compounds from the EO.

### 2.4. Micro-Characterization of the Scaffolds

**Figure 3:**
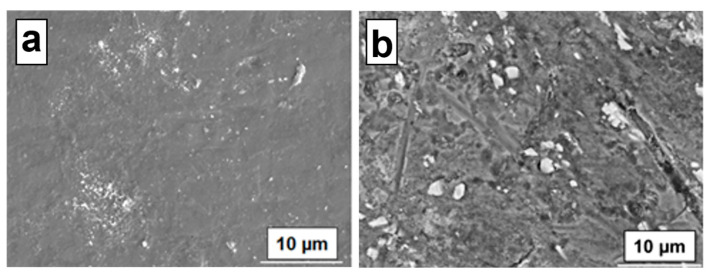
SEM images of the (**a**) PVA–pullulan-0, and (**b**) PVA–pullulan-1.

**Figure 4:**
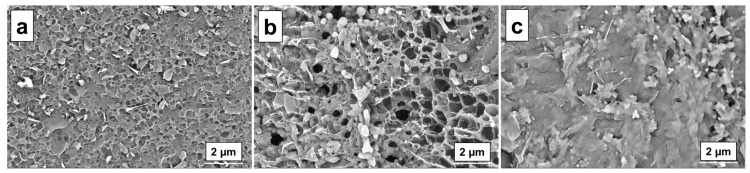
SEM images of the (**a**) PVA–pullulan-0′, (**b**) PVA–pullulan-1′, and (**c**) PVA–pullulan-2′.

The morphology of PVA–pullulan fractured surfaces was investigated by scanning electron microscopy (SEM) (Figure 3 and Figure 4). Figure 3a shows that the surface of the PVA–pullulan-0′ is relatively smooth and dense. The addition of *N. racemosa* EO (Figure 3b) changes the microstructures of the unloaded PVA–pullulan-0 (Figure 3a). As indicated in Figure 3a, unloaded PVA–pullulan-0 had a smooth surface, one with no cracks. In contrast, the addition of *N. racemosa* EO into the polymeric matrix induced the formation of microstructures, some knobs, and a rougher surface in the case of the EO-loaded films, as can be observed in Figure 3b; this might suggest the migration of the extracts from the polymer matrix [54,55].

The SEM images of the cross-sections of the scaffolds based on PVA–pullulan crosslinked with oxalic acid (PVA–pullulan-0′) and PVA–pullulan–oxalic acid loaded with *N. racemosa* EO (PVA–pullulan-1′, PVA–pullulan-2′, respectively) are shown in Figure 4. The PVA–pullulan-0′ has a porous structure with uniform distribution of the symmetrical pores. Nevertheless, increasing further the amount of *N. racemosa* EO loaded into the PVA–pullulan–oxalic acid polymeric matrix resulted in a compact morphology of the material (Figure 4c).

**Figure 5:**
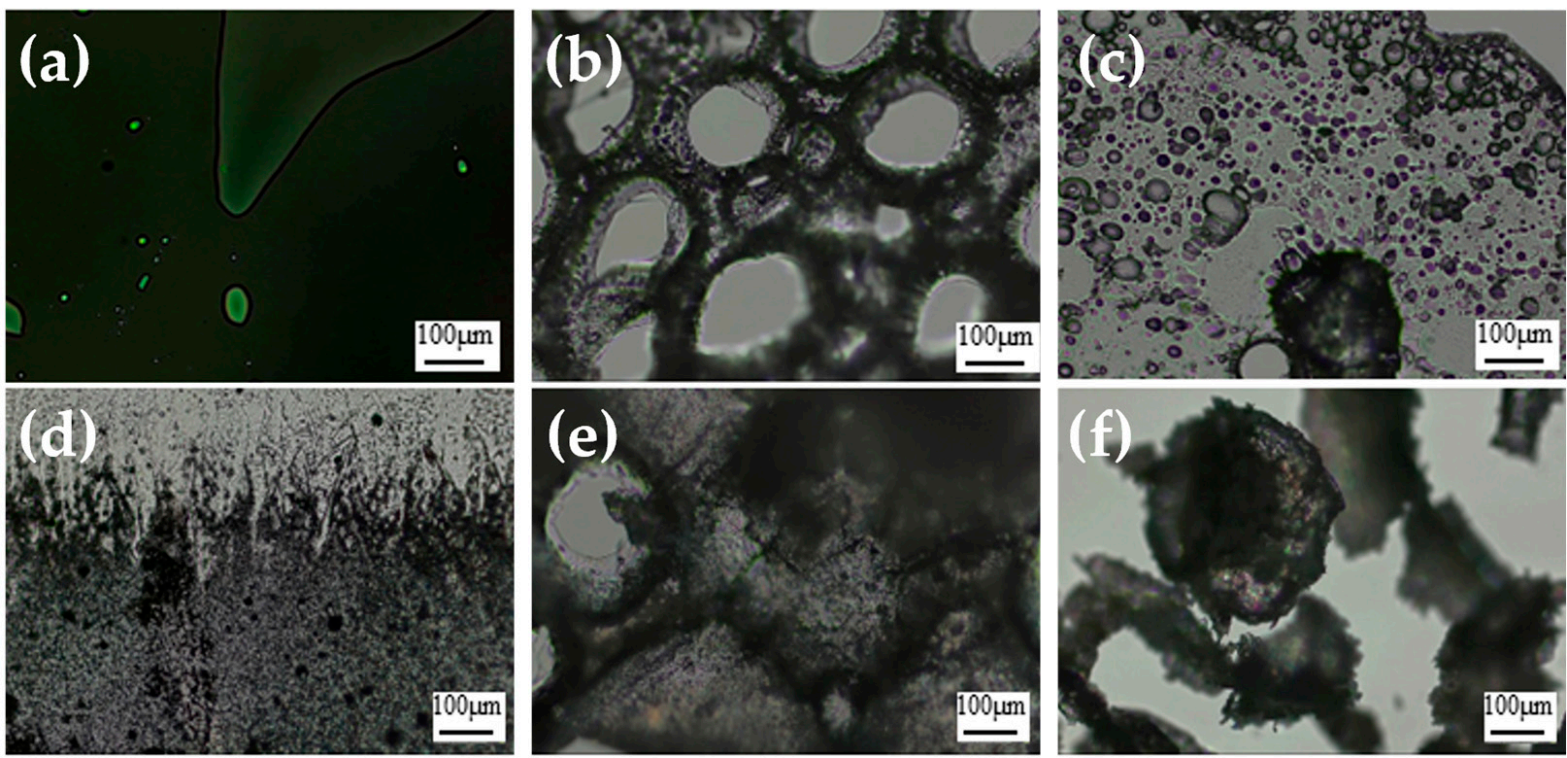
Polarized optical microscopy images of the (**a**) *N. racemosa* EO, (**b**) PVA–pullulan-0, (**c**) PVA–pullulan-1, (**d**) PVA–pullulan-0′, (**e**) PVA–pullulan-1′, and (**f**) PVA–pullulan-2′.

To inspect the appearance of the microscale morphology of the studied samples, polarized light microscopy was used. The samples were subjected to optical microscopy investigation at room temperature and the images taken are presented in Figure 5. *N. racemosa* EO displayed isotropic phases under observed conditions (Figure 5a). 

The inspection of the PVA–pullulan film free of essential oils revealed an interesting morphology with uniformly distributed holes (Figure 5b), which nevertheless collapsed when *N. racemosa* EO was subsequently introduced into the polymeric matrix (Figure 5c). In the latter case, the morphology became more compact and the holes were replaced by droplets with dimensions ranging from 10 to 40 microns, which are suggestive of a uniform distribution of the volatile organic compounds—which exist in the *N. racemosa* EO—across the PVA–pullulan films.

The semi-interpenetrating network effect in the case of samples crosslinked with oxalic acid PVA–pullulan-0′ and the PVA–pullulan–oxalic acid samples loaded with *N. racemosa* EO, PVA–pullulan-1′, and PVA–pullulan-2′, respectively, was revealed by the microimaging of the observed samples. Thus, in the case of the PVA–pullulan-0′ sample, the porous morphology of the polymeric network is presented in the bottom part of Figure 5d, while by inspecting a thinner part of the sample (upper section of the image presented in Figure 5d, some birefringence could be observed in the material, suggesting some non-H-bonded free molecules of oxalic acid in the matrix. By introducing the *N. racemosa* EO in the polymeric matrix, the gel-forming character was even more pronounced, and some aggregates could be observed, suggesting a higher viscosity of the material [56], while the birefringence was not only preserved but even more intense under the focalization of the microscopic view.

### 2.5. Antibacterial Activity of Nepeta racemosa Lam. Essential Oil

**Figure 6:**
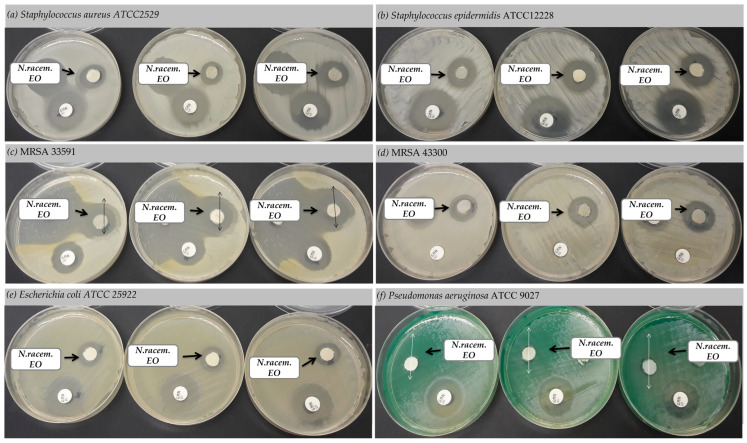
Inhibition zones showing the antibacterial activity of *N. racemosa* EO against some bacterial strains indicated on the figure.

The results showed a good antimicrobial activity of *N. racemosa* EO against gram-positive strains, namely *Staphylococcus aureus* ATCC 25293 (12.33 ± 0.71 mm) and *Staphylococcus epidermidis* ATCC 12228 (20 ± 0.49 mm), but less active compared to the gentamicin control that created an inhibition zone of 20 ± 0.57 mm and 30 ± 0.5 mm, respectively, as can be seen in the data presented in Table 2, and from the inactivation zones shown in Figure 6. One noteworthy aspect is that the antimicrobial activity of this essential oil was superior to that of gentamicin (positive control) in the case of MRSA ATCC 33591 (19 ± 0.75 mm, compared to 13.66 ± 0.43 mm) and MRSA ATCC 43300 (12.66 ± 0.45 mm), where gentamicin was ineffective.

As for the sensitivity of gram-negative bacterial species to *Nepeta racemose*, essential oils showed different results. Thus, the best antimicrobial effect was found on *Nepeta racemosa* essential oils against both *Escherichia coli* (11.33 ± 0.22 mm) and especially *Pseudomonas aeruginosa* (28 ± 0.4 mm), whose zone of inhibition was better compared to the control substance, gentamicin, with 20 ± 0.57 mm and 20 ± 0.5 mm, respectively.

**Figure 7:**
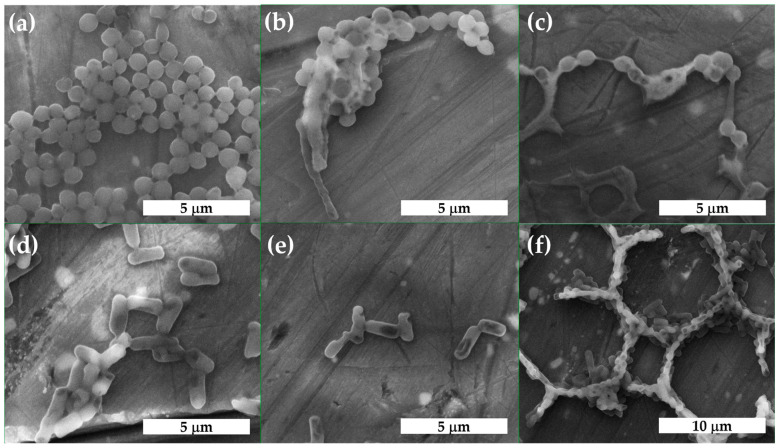
SEM micrographs of *Staphylococcus aureus*: (**a**) untreated, and (**b**,**c**) exposed to *Nepeta racemosa* essential oil; and *Escherichia coli*: (**d**) untreated, and (**e**,**f**) exposed to *Nepeta racemosa* essential oil.

Scanning electron microscopy was used to study the morphology of gram-positive and gram-negative bacterial cells after exposure to *N. racemosa* EO. Analysis of the images clearly showed the significant morphological deterioration of treated cells of *Staphylococcus aureus* and *Escherichia coli* compared to untreated cells, as can be seen in Figure 7. Bacterial cell lysis (Figure 7b,c,e) and agglutination (Figure 7c,f) were observed in both species.

### 2.6. Antibacterial Activity of PVA–Pullulan Fims and Gels Loaded with Nepeta racemosa Lam. Essential Oil

Different amounts (0.1 mL and 0.05 mL) of *Nepeta racemosa* essential oil incorporated in the polyvinyl alcohol (PVA) and pullulan matrix were tested for their antibacterial potential on both gram-positive (*Staphylococcus aureus*) and gram-negative (*Escherichia coli* and *Pseudomonas aeruginosa*) bacteria. For the interpretation, the results were compared to the control represented only by the matrix (PVA–pullulan-0), with an inhibition zone of 5 mm. The data is presented in Table 3. Similarly, as in the case of the films, different amounts (0.1 mL and 0.15 mL) of *N. racemosa* EO were loaded in gels (PVA–pullulan-1′ and PVA–pullulan-2′) and their antibacterial potential was tested on gram-positive (*Staphylococcus aureus*) and gram-negative (*Escherichia coli* and *Pseudomonas aeruginosa*) bacteria. For the interpretation, the results were compared with the control represented only by the matrix (PVA–pullulan-0) and the gel (PVA–pullulan-0′), respectively, both with an inhibition zone of 5 mm. The data is presented in Table 3.

**Figure 8:**
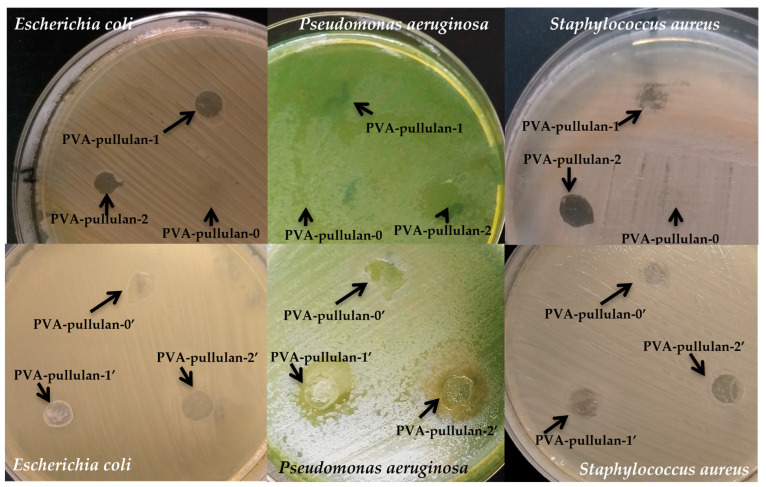
Antibacterial activity of PVA–pullulan films (upper side) and gels (lower side) loaded with *N. racemosa* EO against *Escherichia coli, Pseudomonas aeruginosa*, and *Staphylococcus aureus*.

PVA–pullulan films enhanced with *Napeta racemosa* essential oil showed variable efficiency related to the concentrations of the essential oils. Accordingly, PVA–pullulan-1 inhibited the growth of *Staphylococcus aureus* (10 mm), while PVA–pullulan-2 showed a similar effect to the control. The same activity was observed for *Escherichia coli*, the antimicrobial effect being active in the case of PVA–pullulan-1, with an inhibition zone of 8 mm. In the case of *Pseudomonas aeruginosa*, PVA–pullulan-1 and PVA–pullulan-2 showed inhibition zones of 19 mm and 9 mm, respectively, as presented in Figure 8.

PVA–pullulan gels enriched with *N. racemosa* EO demonstrated antimicrobial activity, inhibiting the multiplication of tested bacterial strains. *Staphylococcus aureus* culture showed inhibition zones of 7 mm (PVA–pullulan-1′) and 9 mm (PVA–pullulan-2′), proving the ability of the active biomolecules in the oils to diffuse into the environment and demonstrating their antimicrobial potential, as presented in Table 3. Testing the effect on *Escherichia coli* showed inhibition zones of 8 mm (PVA–pullulan-1′) and 10 mm (PVA–pullulan-2′). Very good results were obtained in the tests on *Pseudomonas aeruginosa,* with an inhibition zone of about 10 mm in the case of PVA–pullulan-1′, and 17 mm in the case of PVA–pullulan-2′ (Figure 8).

## 3. Discussion

*Nepeta racemosa* is one of more than 280 species of the *Nepeta* genus, the majority being aromatic plants. Scientific studies on *Nepeta* essential oils have reported that a multitude of biological activities arising from its antioxidant, anti-inflammatory, analgesic, anti-depressant, and insecticide properties are being frequently used in traditional therapies in various parts of the world [57,58]. Surprisingly, even fewer details are known about *Nepeta racemosa*, and especially about its antimicrobial properties. Some studies mention the antifungal potential of *Nepeta racemosa* extracts against *Penicillium* and *Aspergillus* fungi, moderate antibacterial activity against *Bacillus anthracis* and *Streptococcus pyogenes,* and the absence of antimicrobial effect against *Escherichia coli* and *Enterococcus faecalis* [59]. The biological properties of *Nepeta* species are usually based on the synergistic action of the active compounds. Many pathogenic bacteria such as *Staphylococcus aureus*, MRSA, *Escherichia coli,* and *Pseudomonas aeruginosa* have a cellular structure that allows them to adhere to biotic and abiotic surfaces and form biofilms, which is considered a protection mechanism that ensures their survival, and increases their virulence and resistance to antibiotics [60]. 

Regarding the extraction of EO, the literature reports variable amounts of volatile oil in the plants. In Iran 0.2% was reported [61], while in Türkiye, 0.7% was found in plants from Erzurum, and 0.11% in those from Kars [62]. A comparative study performed in the USA found 0.07% of oil in *N. racemosa* Lam. in a hybrid culture [63]. The essential oil amount and composition are variable and depend on the plant species, geographical region, and plant development stage at the time of harvest [64].

The chemical composition of the EO was determined by GC-MS; firstly, the volatile compounds were separated by gas chromatography, and then each compound was identified based on its retention time and mass spectrum. The results are not in agreement with existing reports. Nepetalic acid is mostly reported as an important component for other *Nepeta* species other than *racemosa* such as *Nepeta cataria* L., *Nepeta caesarea* Boiss., *Nepeta nuda* ssp. albiflora (Boiss.) Gams and *Nepeta teydea* Webb. & Berth. [65]. Detailed studies on nepetalic acid were only performed on *Nepeta cataria* L. due to the fact that this compound has repellant properties for mosquitos and other insects [66]. The second compound, eucalyptol, was found in different amounts from 0.1 to 21% [67,68]. 3-Hydroxy-(4S,4aS,7S,7aS)-Dihydronepetalactone was also found in *Nepeta argolica* ssp. *argolica* Bory & Chaub [69]. It seems as if the biochemical diversity and variability of the compounds depends on many factors, such as chemotype, natural factors, technological factors, and stress factors. 

From the SEM images (Figure 3a) it can be observed that PVA–pullulan have homogenous surfaces; this indicates that PVA and pullulan mixed effectively. By comparing Figure 3a with Figure 4a we can conclude that, in the case of Figure 4a, the internal structure of the construct between PVA crosslinked with oxalic acid and pullulan exhibited a highly porous morphology with irregular patterns and also displayed interconnected pores. This means that the crosslinking of PVA with oxalic acid led to the formation of micro and nano-scaled pores in the PVA–pullulan-0′ sample compared with the PVA–pullulan-0 sample. Thus, the presence of pores in the PVA–pullulan-0′ makes this scaffold more efficient for loading different bioactive compounds. In this respect, and to water-stabilize the hydrophilic PVA, oxalic acid was used as a physical crosslinker. Some generally spherical particles can be observed in the PVA–pullulan-1 (Figure 3b) and PVA–pullulan-1′ (Figure 4b). However, a slight increase in the degree of agglomeration of the generally spherical particles onto the PVA–pullulan-2′ can be also observed, giving them a slightly irregular and enlarged form (Figure 4c).

*Nepeta racemosa* EO showed great antimicrobial activity on several gram-negative and gram-positive strains. Simultaneous testing on both bacterial types was necessary, since both have different cell wall structures and varying affinities for gram strain. Structurally, gram-positive bacteria are surrounded by a single thick peptidoglycan cell wall, while the gram-negative have a much thinner peptidoglycan cell wall and an outer membrane containing lipopolysaccharides. SEM imaging of the bacteria after treatment with *N. racemosa* EO showed cell membrane deterioration, diffusion, and bacterial cell lysis, as well as agglutination for both *Staphylococcus aureus* and *Escherichia coli*, as indicated in Figure 7. Similar results have been reported for *S. epidermidis* under the action of *Zanthoxylum schinifolium* EO; it is thought that the cell membrane permeability was affected, and that this resulted in the lysis of the cell wall and the leakage of intracellular constituents [70]. Other pathogens have exhibited the same behavior under the exposure to *Helichrysum italicum* (Roth) G. Don EO [71]. The antimicrobial activity of essential oils extracted from *Nepeta racemosa* might be due to the presence of bioactive phytocompounds such as 4a-α,7-α,7a-β-nepetalactone and 4a-α,7-β,7a-α-nepetalactone, which, in association with krypton, α-pinene, isolimonene, and caryophyllene oxide, determine the antimicrobial activity. The antimicrobial activity is expressed by the synergistic action of chemical compounds involved in microbial inhibition and distribution through different mechanisms [72]. 

The introduction of *N. racemosa* EO resulted in a semi-interpenetrated polymer network revealed by the formation of larger and non-oriented pores, while the pore distribution is similar to a sponge network, as indicated by the SEM imaging [73]. The PVA–pullulan formulation showed both open and closed pore structures and an increased interconnection between pores carrying the phytotherapeutic agents. On the entire microscopic view, no droplets were observed in the case of the *N. racemosa* EO sample, indicating a higher purity of the essential oil. In some situations, tiny droplets can indicate the presence of residual vegetal matter or some other artifacts or impurities resulting from the production of the essential oil [74]. 

Except for the tests regarding the antimicrobial activity of EO, our study focused on obtaining two matrices based on PVA–pullulan as a solid support and carrier for the essential oils. It is not enough to prove that the essential oils can be retained by the PVA–pullulan films and gels; the most important question being their ability to release the active compounds having antimicrobial activity. Both enriched films and gels showed promising activity against gram-positive and gram-negative bacteria. Encouraging results were obtained when testing the antibacterial activity against MRSA strains that showed resistance to the antibiotics or reduced sensibility. This aspect is particularly important, because methicillin-resistant staphylococci, along with other antibiotic-resistant microorganisms, pose a special challenge in current medical practice. MRSA have a remarkable ability to adapt to different environments and hosts and have evolved over time into epidemic strains that pose a major public health risk [75].

PVA–Pullulan films were loaded with *N. racemosa* EO, as the gels had been, to test their antibacterial activity against gram-positive bacteria (*Staphylococcus aureus*). It was observed that the best effect was obtained with PVA–pullulan-1. In the case of gram-negative bacteria, the most sensitive proven to be *Pseudomonas aeruginosa*, which was more sensitive than *Escherichia coli*. Its strong performance as to this gram-negative species can be explained by the combined action of the active principles in the essential oil, which offers a possible alternative therapy in the context of infections produced by this microorganism, considering its role as a potential pathogen for humans and animals and its multiple antibiotic resistance. For the PVA–pullulan *N. racemosa* EO-loaded gels, the EO proved to have a superior effect on gram-negative bacteria, especially against *Pseudomonas aeruginosa.* These results indicate the possibility of using both gels and films loaded with *N. racemosa* EO for various applications. Currently, polymers obtained from natural sources are trying to replace synthetic polymers for their applications in the medical or agri-food fields. Based on the literature data regarding the matrix components used in this study, it can be considered a biodegradable polymer [76,77]. The edible films, enriched with essential oils, offer improved functionality and can be used for active packaging of perishable food products (meat, fish, seafood, fruits, vegetables, etc.), increasing their shelf life by preventing microbial spoilage [52,53]. Also, polymers with antibacterial activity could be used to prevent the formation of microbial biofilms. Some studies have also shown that the polysaccharides used in the composition of the matrix where the essential oils were included can also contribute to the final antimicrobial effect of the functional complex; the hydroxyl groups present on the PVA molecules and some residual carboxyl from the chemical crosslinker could also have some inhibitory effect [78].

Hydrogels have a physical structure that allows them to be displayed on different animated surfaces, and the improvement of these matrices using essential oils with proven antimicrobial activity offers the possibility of using them for skin conditions through topical applications with a curative purpose, either to treat infections or prophylactically to prevent contamination of wounds or skin lesions. Studies have already been carried out with hydrogels in which essential oils of lavender, tea tree, thyme, rosemary, oregano, cinnamon, etc. have been incorporated, having as its main use the treatment of infected wounds. A similar study refers to the use of PVA/starch hydrogel membranes enriched with clove oil, tea tree oil, and oregano oil for wound dressing [79]. The functionalized hydrogels can retain or encapsulate essential volatile oils and can be used to control bacteria multiplication by a gradual release of the active compounds in the target area. For the same purpose, those active systems can be used to control or treat viral and fungal infections. 

## 4. Materials and Methods

### 4.1. Plant Collection and Extraction

The plants were taken from the Collection of Medicinal and Aromatic Plants of the Iasi University of Life Sciences in northeastern Romania (47°11′27.5″ N, 27°33′17.9″ E). *Nepeta racemosa* is not a wild species in our country, the plants being grown in controlled culture. The extraction of the essential oils was performed through hydro-distillation in a high-capacity Clevenger apparatus. A quantum of 1000 g of fresh material, composed of aerial parts of the plants, was boiled for 3 h. The oil was collected and deposited in opaque vials at 4 °C. 

### 4.2. GC-MS Analysis

The chemical composition of the volatile oils was established by gas-chromatography combined with mass spectrometry using an Agilent Technologies 6890N chromatograph coupled (Agilent Technologies, Palo Alto, USA) with the mass detector 5975 inert XL Mass Selective Detector. For GC, a column DB5 with an external diameter of 30 m × 0.25 mm and internal diameter of 0.25 μm (5% Phenylmethylsiloxane) was used with helium as mobile phase at a flow rate of 1mL/min, injector temperature 220 °C, detector temperature 250 °C, a temperature regime of 60 °C initial temperature with an increasing rate of 3 degrees per minute up to 246 °C and then kept constant for 8 min, injected volume 0.1–0.3 μL, and split rate 1:100. The chromatographic peaks were identified based on the NIST 2008 database and confirmed by mass spectrum and retention time [80].

### 4.3. Preparation of the Films and Gels

PVA powder with average molecular weights M_w_ = 30,000–70,000 Da and a degree of hydrolysis of 87–90% was purchased from Sigma-Aldrich (Sigma-Aldrich Chemie GmbH, Eschenstraße 5, 82024 Taufkirchen, Germany). Pullulan powder was purchased from TCI EUROPE (Zwijndrecht, Belgium). Oxalic acid was purchased from Merck (Darmstadt, Germany). All other reagents were used as received from commercial sources or were purified by standard methods.

The PVA solution was prepared by dissolving a fixed amount of PVA powder by weight in distilled water at high temperature under constant stirring for 2 h to obtain a solution with a concentration of 10% wt./v. The same procedure was used for the pullulan solution, only the concentration was 5% wt./v. To obtain the pristine solution (PVA–pullulan-0), 1.6 mL PVA solution and 0.4 mL pullulan solution were mixed under stirring for 1 h at room temperature. The resulting homogeneous PVA–pullulan solution was then cast on glass plates to obtain the PVA–pullulan-0 film. For the samples denoted as PVA–pullulan-1 and PVA–pullulan-2, 0.1 mL *N. racemosa* EO, and 0.05 mL *N. racemosa* EO, respectively, were added to the pristine solution of PVA–pullulan-0. These mixtures were stirred at room temperature for 4 h to ensure that the EO was well dispersed in the PVA–pullulan composite solution. The prepared solutions (PVA–pullulan loaded with EO) were sonicated for 30 min with an ultrasonic cleaner to remove bubbles (Model FB11012, Fisherbrand, Loughborough, UK). Following that, the mixtures (2 mL) were cast in different Petri dishes and dried for 2 h at 50 °C. Before characterization tests, the films were stored at a constant temperature (25 °C) in a humidity chamber for at least two days [81]. In Table 4 full data relating to the composition of PVA–pullulan film samples are listed.

**Scheme 1:**
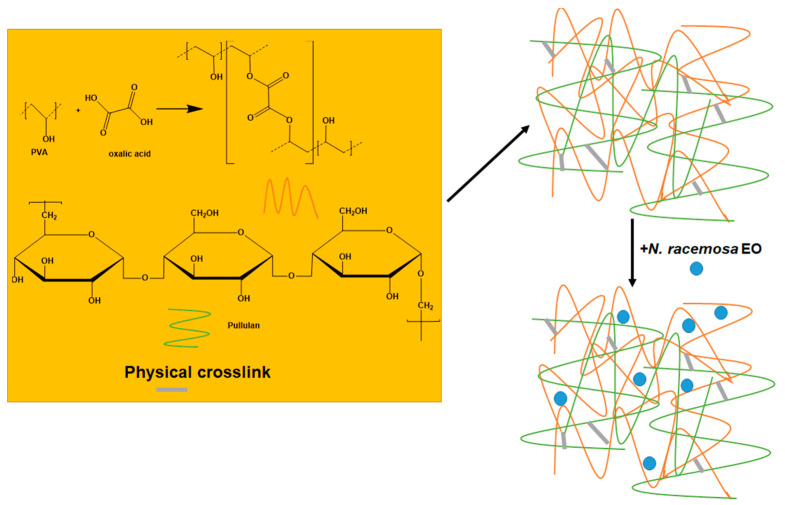
Schematic illustration of the preparation of PVA–pullulan gels loaded with EO.

To obtain network-like PVA–pullulan scaffolds, the pristine solution PVA–pullulan-0 was crosslinked with oxalic acid. The sample denoted as PVA–pullulan-0′ was prepared by mixing 1.6 mL PVA solution with 0.4 mL pullulan solution and 0.1 g oxalic acid. After complete dissolution, the mixture was poured into a container and placed in the oven at a temperature of 60 °C for 1 h. For the samples denoted as PVA–pullulan-1′ and PVA–pullulan-2′, 0.1 mL *N. racemosa* EO and 0.15 mL *N. racemosa* EO were added to PVA–pullulan-0′ solution. The same procedure as in the case of the PVA–pullulan-0′ gel was used for these gels containing *N. racemosa* EO in their structure (Scheme 1). The details of the samples are shown in Table 5. A schematic representation depicting the synthetic pathways of the PVA–pullulan gels loaded with *N. racemosa* EO is shown in Scheme 1.

### 4.4. Characterization of the Films and Gels

Optical microscopy images were taken on samples at ambient temperature, using a Zeiss Microscope Axio Imager A2M equipped with Linkam Plate LTS420 with a 10× objective (Carl Zeiss Microscopy GmbH, Oberkochen, Germany).

A Verios G4 UC Scanning Electron Microscope (Thermo Scientific, FEI, Brno, Czech Republic) was used to investigate the surface morphology of the loaded scaffolds. To this purpose, the samples were coated with 6 nm platinum using a Leica EM ACE200 sputter coater (Leica Microsystems, Vienna, Austria) to provide electrical conductivity and prevent charge buildup during exposure to the electron beam. SEM investigations were performed in High Vacuum mode using a secondary electron detector (Everhart-Thornley detector, ETD) at an accelerating voltage of 10 kV [82].

UV-VIS spectra of *N. racemosa* EO and *N. racemosa* released from PVA–pullulan-1′ and PVA–pullulan-2′ gels were recorded using a UV spectrophotometer (SPECORD 21 Plus Analytik, Jena, Germany). The interval for scans was in the spectral range of 200–500 nm. The test was repeated three times for each sample.

For the identification of the chemical structure of the investigated samples a LUMOS Microscope Fourier Transform Infrared (FTIR) spectrophotometer (Bruker Optik GmbH, Ettlingen, Germany), equipped with an attenuated total reflection (ATR) device was used at wave numbers ranging from 4000 to 500 cm^−1^ at a resolution of 4 cm^−1^.

### 4.5. Assessment of the Antibacterial Analysis

The antimicrobial activity of *N. racemosa* EO was tested on six standardized bacterial strains (ATCC); five of them were gram-positive, namely, *Staphylococcus aureus* ATCC 25293, methicillin-resistant *Staphylococcus aureus* (MRSA) ATCC 33591, methicillin-resistant *Staphylococcus aureus* (MRSA) ATCC 43300, *Staphylococcus epidermidis* ATCC 12228; additionally, two gram-negative strains were used, namely, *Escherichia coli* ATCC 25922 and *Pseudomonas aeruginosa* ATCC 9027.

All bacterial strains were cultivated in Mueller-Hinton broth (Oxoid) and incubated for 24 h. Microbial suspensions with a density corresponding to a turbidity of 0.5 on the McFarland standard nephelometric scale were prepared (0.5 × 10^8^ CFU) and 1 mL of each bacterial strain culture was spread on the surface of Mueller-Hinton agar distributed in a sterile Petri dish. In order to test the antimicrobial activity of the EO, sterile filter paper discs (Φ5 mm) were placed on the surface of the cultivated agar as a support for 10 µL of *N. racemosa* EO. A gentamicin disc (10 µg/Φ 5 mm) was used as a positive control. After incubation at 37 °C for 24h, the antimicrobial activity was evaluated by measuring the diameter of bacteria inhibition zones. The results obtained in the essential oils test were compared with those of the positive control (gentamicin).

*Staphylococcus aureus* and *Escherichia coli* cell morphology after *Nepeta racemosa* essential oil treatment was evaluated using the SEM method. Microbial suspensions (1 mL) brought to a cell density of 1.5 × 10^8^ cfu/mL were exposed to *Nepeta racemosa* essential oil (10 µL) for 24 h. The cell control was represented by untreated bacterial strains. 10 μL of each was dropped on the sterile aluminum stubs used as support for environmental scanning electron microscopy. Quanta 450 (FEI, Thermo Fisher Scientific, Hillsboro, OR, USA) was used in low vacuum mode with an electron beam at 15 kV to image the samples. 

The antimicrobial activity of the films and gels was determined for three bacterial strains (*Escherichia coli* ATCC 25922, *Pseudomonas aeruginosa* ATCC 9027, and *Staphylococcus aureus* ATCC 25293) using the same disk diffusion Kirby-Bauer method adapted for testing natural polymer matrices. Under aseptic conditions, the PVA–pullulan films and the gels samples with (PVA–pullulan-1, PVA–pullulan-2, PVA–pullulan-1′, PVA–pullulan-2′) and without *N. racemosa* EO (PVA–pullulan-0, PVA–pullulan-0′) were punched in the form of discs with a diameter of 5 mm and displayed on the surface of the Mueller Hinton agar inoculated with bacterial culture (*Staphylococcus aureus, Escherichia coli* and *Pseudomonas aeruginosa*). The evaluation of the antimicrobial activity of the polymers enriched with *Nepeta racemosa* essential oils was performed by measuring the diameter of the inhibition zones, and comparing the results with those from the negative control (PVA–pullulan-0 for the films, and PVA–pullulan-0′, for the gels).

## 5. Conclusions

In this work, we focused on the antimicrobial properties of *Nepeta racemosa* Lam. essential oil and the possibility of incorporating it in applications. We showed that, in our case, the most important component of the essential oil is the nepetalic acid. *Nepeta racemosa* EO showed very good antimicrobial activity against gram-positive and gram-negative strains, inactivating them as proven by the evaluation of the inhibition zones of cultures treated with EO and SEM measurements as well. The latter indicated that EO produces significant deterioration of the bacterial cell wall and agglutination. 

Both films and gels loaded with *N. racem.* EO had good antibacterial properties against some bacterial strains, showing the possibility of using these structures in food packaging or biomedical applications. Our future interest regards not only the actual applications of materials loaded with *Nepeta racemosa* EO based on their antimicrobial activity, but also studying the extracted oils from other *Nepeta* sp. in an attempt to enhance their antimicrobial activity. 

## Figures and Tables

**Table 1:** Chemical composition of *Nepeta racemosa* Lam. essential oil, from GC-MS analysis.

Number	RT (min)	Indics Kovats	Compounds	*Nepeta racemosa* Lam.
1.	5.85	930	α-Pinene	0.4
2.	7.03	978	β-Pinene	0.9
3.	7.19	985	3-Octanone	0.3
4.	7.39	993	β-Myrcene	0.2
5.	8.74	1032	**Eucalyptol**	**10.7**
6.	9.31	1048	cis-β-Ocimene	1.1
7.	11.20	1101	Linalol	0.9
8.	14.35	1180	4-Terpineol	0.4
9.	17.67	1258	Linalyl acetate	0.3
10.	21.96	1359	(4aS,7S,7aS)-trans,cis-Nepetalactone	5.0
11.	22.77	1378	α-Copaene	1.9
12.	23.14	1387	(4aS,7S,7aR)-cis,trans-Nepetalactone	1.0
13.	24.57	1422	β-Caryophyllene	3.7
14.	25.92	1455	α-Humulene	0.5
15.	27.03	1483	Germacren D	4.7
16.	27.38	1491	**3-Hydroxy-(4S,4aS,7S,7aS)-** **Dihydronepetalactone**	**5.4**
17.	28.70	1525	δ-Cadinene	1.5
18.	28.96	1532	**Nepetalic acid**	**55.5**
19.	30.95	1583	Spathulenol	0.3
20.			Other compounds	5.3

**Table 2:** The antibacterial activity of *Nepeta racemosa* Lam. essential oil against some bacterial strains.

Bacterial Strains		Mean Diameters of Inhibition Zones (mm)	
		Gentamicin 10 µg/Φ5 mm	*N. racemosa* EO 10 µL/Φ5 mm
Gram-positive	*S. aureus* ATCC 25293	20 ± 0.57	12.33 ± 0.71
	MRSA ATCC 33591	13.66 ± 0.43	19 ± 0.75
	MRSA ATCC 43300	0	12.66 ± 0.45
	*S. epidermidis* ATCC12228	30 ± 0.50	20 ± 0.49
Gram-negative	*E.coli* ATCC 25922	20 ± 0.57	11.33 ± 0.22
	*P. aeruginosa* ATCC 9027	20 ± 0.50	28 ± 0.40

**Table 3:** The antibacterial activity of PVA–pullulan films and gels loaded with *Nepeta racemosa* Lam. essential oil.

Sample Type	*Staphylococcus aureus* (Gram-Positive)	*Escherichia coli* (Gram-Negative)	*Ps. aeruginosa* (Gram-Negative)
disc Ø 5 mm	Ø mm	Ø mm	Ø mm
PVA–pullulan-0 (control)	5 ± 0.25	5 ± 0.43	5 ± 0.29
PVA–pullulan-1 (0.1 mL *N. racem.* EO)	10 ± 0.51	8 ± 0.53	19 ± 0.75
PVA–pullulan-2 (0.05 mL *N. racem.* EO)	5 ± 0.29	5 ± 0.59	9 ± 0.51
PVA–pullulan-0′ (control)	5 ± 0.18	5 ± 0.50	5 ± 0.43
PVA–pullulan-1′ (0.1 mL *N. racem.* EO)	7 ± 0.41	8 ± 0.33	10 ± 0.45
PVA–pullulan-2′ (0.05 mL *N. racem.* EO)	9 ± 0.55	10 ± 0.25	17 ± 0.41

**Table 4:** The composition of PVA–pullulan film samples.

Sample Code	PVA (g)	Pullulan (g)	*N. racemosa* EO (mL)
PVA–pullulan-0	0.1777	0.0211	-
PVA–pullulan-1	0.1777	0.0211	0.1
PVA–pullulan-2	0.1777	0.0211	0.05

**Table 5:** The composition of PVA–pullulan gel samples.

Samples Code	PVA (g)	Pullulan (g)	Oxalic Acid (g)	*N. racemosa* EO (mL)
PVA–pullulan-0′	0.1777	0.0211	0.1	-
PVA–pullulan-1′	0.1777	0.0211	0.1	0.1
PVA–pullulan-2′	0.1777	0.0211	0.1	0.15

## Data Availability

Not applicable.
